# Association between problematic social networking use and anxiety symptoms: a systematic review and meta-analysis

**DOI:** 10.1186/s40359-024-01705-w

**Published:** 2024-05-12

**Authors:** Mingxuan Du, Chengjia Zhao, Haiyan Hu, Ningning Ding, Jiankang He, Wenwen Tian, Wenqian Zhao, Xiujian Lin, Gaoyang Liu, Wendan Chen, ShuangLiu Wang, Pengcheng Wang, Dongwu Xu, Xinhua Shen, Guohua Zhang

**Affiliations:** 1 School of Mental Health, Wenzhou Medical University, 325035 Wenzhou, China; 2 School of Education, Renmin University of China, 100872 Beijing, China; 3 School of Media and Communication, Shanghai Jiao Tong University, Dongchuan Road 800, 200240 Shanghai, China; 4 Department of Neurosis and Psychosomatic Diseases, Huzhou Third Municipal Hospital, 313002 Huzhou, China

**Keywords:** Problematic social networking use, Generalized anxiety, Social anxiety, Attachment anxiety, Fear of missing out, Meta-analysis

## Abstract

A growing number of studies have reported that problematic social networking use (PSNU) is strongly associated with anxiety symptoms. However, due to the presence of multiple anxiety subtypes, existing research findings on the extent of this association vary widely, leading to a lack of consensus. The current meta-analysis aimed to summarize studies exploring the relationship between PSNU levels and anxiety symptoms, including generalized anxiety, social anxiety, attachment anxiety, and fear of missing out. 209 studies with a total of 172 articles were included in the meta-analysis, involving 252,337 participants from 28 countries. The results showed a moderately positive association between PSNU and generalized anxiety (GA), social anxiety (SA), attachment anxiety (AA), and fear of missing out (FoMO) respectively (GA: *r* = 0.388, 95% *CI* [0.362, 0.413]; SA: *r* = 0.437, 95% *CI* [0.395, 0.478]; AA: *r* = 0.345, 95% *CI* [0.286, 0.402]; FoMO: *r* = 0.496, 95% *CI* [0.461, 0.529]), and there were different regulatory factors between PSNU and different anxiety subtypes. This study provides the first comprehensive estimate of the association of PSNU with multiple anxiety subtypes, which vary by time of measurement, region, gender, and measurement tool.

## Introduction

Social network refers to online platforms that allow users to create, share, and exchange information, encompassing text, images, audio, and video [1]. The use of social network, a term encompassing various activities on these platforms, has been measured from angles such as frequency, duration, intensity, and addictive behavior, all indicative of the extent of social networking usage [2]. As of April 2023, there are 4.8 billion social network users globally, representing 59.9% of the world’s population [3]. The usage of social network is considered a normal behavior and a part of everyday life [4, 5]. Although social network offers convenience in daily life, excessive use can lead to PSNU [6, 7], posing potential threats to mental health, particularly anxiety symptoms (Rasmussen et al., 2020). Empirical research has shown that anxiety symptoms, including generalized anxiety (GA), social anxiety (SA), attachment anxiety (AA), and fear of missing out (FoMO), are closely related to PSNU [8–12]. While some empirical studies have explored the relationship between PSNU and anxiety symptoms, their conclusions are not consistent. Some studies have found a significant positive correlation [13–15], while others have found no significant correlation [16–19]. Furthermore, the degree of correlation varies widely in existing research, with reported r-values ranging from 0.12 to 0.80 [20, 21]. Therefore, a systematic meta-analysis is necessary to clarify the impact of PSNU on individual anxiety symptoms.

Previous research lacks a unified concept of PSNU, primarily due to differing theoretical interpretations by various authors, and the use of varied standards and diagnostic tools. Currently, this phenomenon is referred to by several terms, including compulsive social networking use, problematic social networking use, excessive social networking use, social networking dependency, and social networking addiction [22–26]. These conceptual differences hinder the development of a cohesive and systematic research framework, as it remains unclear whether these definitions and tools capture the same underlying construct [27]. To address this lack of uniformity, this paper will use the term “problematic use” to encompass all the aforementioned nomenclatures (i.e., compulsive, excessive, dependent, and addictive use).

Regarding the relationship between PSNU and anxiety symptoms, two main perspectives exist: the first suggests a positive correlation, while the second proposes a U-shaped relationship. The former perspective, advocating a positive correlation, aligns with the social cognitive theory of mass communication. It posits that PSNU can reinforce certain cognitions, emotions, attitudes, and behaviors [28, 29], potentially elevating individuals’ anxiety levels [30]. Additionally, the cognitive-behavioral model of pathological use, a primary framework for explaining factors related to internet-based addictions, indicates that psychiatric symptoms like depression or anxiety may precede internet addiction, implying that individuals experiencing anxiety may turn to social networking platforms as a coping mechanism [31]. Empirical research also suggests that highly anxious individuals prefer computer-mediated communication due to the control and social liberation it offers and are more likely to have maladaptive emotional regulation, potentially leading to problematic social network service use [32]. Turning to the alternate perspective, it proposes a U-shaped relationship as per the digital Goldilocks hypothesis. In this view, moderate social networking usage is considered beneficial for psychosocial adaptation, providing individuals with opportunities for social connection and support. Conversely, both excessive use and abstinence can negatively impact psychosocial adaptation [33]. In summary, both perspectives offer plausible explanations.

Incorporating findings from previous meta-analyses, we identified seven systematic reviews and two meta-analyses that investigated the association between PSNU and anxiety. The results of these meta-analyses indicated a significant positive correlation between PSNU and anxiety (ranging from 0.33 to 0.38). However, it is evident that these previous meta-analyses had certain limitations. Firstly, they focused only on specific subtypes of anxiety; secondly, they were limited to adolescents and emerging adults in terms of age. In summary, this systematic review aims to ascertain which theoretical perspective more effectively explains the relationship between PSNU and anxiety, addressing the gaps in previous meta-analyses. Additionally, the association between PSNU and anxiety could be moderated by various factors. Drawing from a broad research perspective, any individual study is influenced by researcher-specific designs and associated sample estimates. These may lead to bias compared to the broader population. Considering the selection criteria for moderating variables in empirical studies and meta-analyses [34, 35], the heterogeneity of findings on problematic social network usage and anxiety symptoms could be driven by divergence in sample characteristics (e.g., gender, age, region) and research characteristics (measurement instrument of study variables). Since the 2019 coronavirus pandemic, heightened public anxiety may be attributed to the fear of the virus or heightened real life stress. The increased use of electronic devices, particularly smartphones during the pandemic, also instigates the prevalence of problematic social networking. Thus, our analysis focuses on three moderators: sample characteristics (participants’ gender, age, region), measurement tools (for PSNU and anxiety symptoms) and the time of measurement (before COVID-19 vs. during COVID-19).

## Method

The present study was conducted in accordance with the 2020 statement on Preferred Reporting Items for Systematic Reviews and Meta-Analyses (PRISMA) [36]. To facilitate transparency and to avoid unnecessary duplication of research, this study was registered on PROSPERO, and the number is CRD42022350902.

### Literature search

Studies on the relationship between the PSNU and anxiety symptoms from 2000 to 2023 were retrieved from seven databases. These databases included China National Knowledge Infrastructure (CNKI), Wanfang Data, Chongqing VIP Information Co. Ltd. (VIP), Web of Science, ScienceDirect, PubMed, and PsycARTICLES. The search strings consisted of (a) anxiety symptoms, (b) social network, and (c) Problematic use. As shown in Table 1, the keywords for anxiety are as follows: anxiety, generalized anxiety, social anxiety, attachment anxiety, fear of missing out, and FoMO. The keywords for social network are as follows: social network, social media, social networking site, Instagram, and Facebook. The keywords for addiction are as follows: addiction, dependence, problem/problematic use, excessive use. The search deadline was March 19, 2023. A total of 2078 studies were initially retrieved and all were identified ultimately.

**Table 1:** Retrieval information

Item			Content							
Academic database			“China National Knowledge Infrastructure (CNKI)”, “WANFANG DATA”, “Chongqing VIP Information Co. Ltd. (VIP)”, “Web of Science”, “ScienceDirect”, “PubMed”, “PsycARTICLES”							
Search elements			(a) Anxiety symptoms: “anxiety” OR “generalized anxiety” OR “social anxiety” OR “attachment anxiety” OR “FoMO” OR “fear of miss out”							
			(b) Social network: “social network”, “social networking site”, “social media”, “Instagram”, “Facebook”							
			(c) Problematic use: “problematic /problem use”, “excessive use”, “addiction”, “dependence”							

### Inclusion and exclusion criteria

Retrieved studies were eligible for the present meta-analysis if they met the following inclusion criteria: (a) the study provided Pearson correlation coefficients used to measure the relationship between PSNU and anxiety symptoms; (b) the study reported the sample size and the measurement instruments for the variables; (c) the study was written in English and Chinese; (d) the study provided sufficient statistics to calculate the effect sizes; (e) effect sizes were extracted from independent samples. If multiple independent samples were investigated in the same study, they were coded separately; if the study was a longitudinal study, they were coded by the first measurement. In addition, studies were excluded if they: (a) examined non-problematic social network use; (b) had an abnormal sample population; (c) the results of the same sample were included in another study and (d) were case reports or review articles. Two evaluators with master’s degrees independently assessed the eligibility of the articles. A third evaluator with a PhD examined the results and resolved dissenting views.

### Data extraction and quality assessment

Two evaluators independently coded the selected articles according to the following characteristics: literature information, time of measurement (before the COVID-19 vs. during the COVID-19), sample source (developed country vs. developing country), sample size, proportion of males, mean age, type of anxiety, and measurement instruments for PSNU and anxiety symptoms. The following principles needed to be adhered to in the coding process: (a) effect sizes were extracted from independent samples. If multiple independent samples were investigated in the same study, they were coded separately; if the study was a longitudinal study, it was coded by the first measurement; (b) if multiple studies used the same data, the one with the most complete information was selected; (c) If studies reported *t* or *F* values rather than *r*, the following formula $$ r=\sqrt{\frac{{t}^{2}}{{t}^{2}+df}}$$; $$ r=\sqrt{\frac{F}{F+d{f}_{e}}}$$ was used to convert them into *r* values [37, 38]. Additionally, if some studies only reported the correlation matrix between each dimension of PSNU and anxiety symptoms, the following formula $$ {r}_{xy}=\frac{\sum {r}_{xi}{r}_{yj}}{\sqrt{n+n(n-1){r}_{xixj}}\sqrt{m+m(m-1){r}_{yiyj}}}$$ was used to synthesize the *r* values [39], where *n* or *m* is the number of dimensions of variable x or variable y, respectively, and$$ {r}_{xixj} $$or $$ {r}_{yiyj}$$ represents the mean of the correlation coefficients between the dimensions of variable x or variable y, respectively.

Literature quality was determined according to the meta-analysis quality evaluation scale developed [40]. The quality of the post-screening studies was assessed by five dimensions: sampling method, efficiency of sample collection, level of publication, and reliability of PSNU and anxiety symptom measurement instruments. The total score of the scale ranged from 0 to 10; higher scores indicated better quality of the literature.

### Data analysis

All data were performed using Comprehensive Meta Analysis 3.3 (CMA 3.3). Pearson’s product-moment coefficient *r* was selected as the effect size index in this meta-analysis. Firstly, ${\text{F}\text{i}\text{s}\text{h}\text{e}\text{r}}^{{\prime }}\text{s} Z=\frac{1}{2}\times \text{ln}\left(\frac{1+r}{1-r}\right)$ was used to convert the correlation coefficient to Fisher *Z*. Then the formula $SE=\sqrt{\frac{1}{n-3}}$ was used to calculate the standard error (*SE*). Finally, the summary of *r* was obtained from the formula $r=\frac{{e}^{2z}-1}{{e}^{2z}+1}$ for a comprehensive measure of the relationship between PSNU and anxiety symptoms [37, 41].

Although the effect sizes estimated by the included studies may be similar, considering the actual differences between studies (e.g., region and gender), the random effects model was a better choice for data analysis for the current meta-analysis. The heterogeneity of the included study effect sizes was measured for significance by Cochran’s *Q* test and estimated quantitatively by the *I*^2^ statistic [42]. If the results indicate there is a significant heterogeneity (the *Q* test: *p*-value < 0.05, *I*^2^ > 75) and the results of different studies are significantly different from the overall effect size. Conversely, it indicates there are no differences between the studies and the overall effect size. And significant heterogeneity tends to indicate the possible presence of potential moderating variables. Subgroup analysis and meta-regression analysis were used to examine the moderating effect of categorical and continuous variables, respectively.

Funnel plots, fail-safe number (Nfs) and Egger linear regression were utilized to evaluate the publication bias [43–45]. The likelihood of publication bias was considered low if the intercept obtained from Egger linear regression was not significant. A larger Nfs indicated a lower risk of publication bias, and if Nfs < 5k + 10 (k representing the original number of studies), publication bias should be a concern [46]. When Egger’s linear regression was significant, the Duval and Tweedie’s trim-and-fill was performed to correct the effect size. If there was no significant change in the effect size, it was assumed that there was no serious publication bias [47].

A significance level of *P* < 0.05 was deemed applicable in this study.

**Table 2:** Characteristics of the selected studies

Name (year)	time of measurement	Country	Simple size	Age	Gender	r	Anxiety symptoms	PSNU scale	Anxiety scale	Literature quality
Al-Mamun et al. [48]	During COVID-19	Developing country	601	NA	0.572	0.460	GA	BSMAS	GAD	7
Andreassen et al. [49]	Before COVID-19	Developed country	23,533	35.8	0.350	0.340	GA	BSMAS	HADS-A	6
Arikan et al. [50]	Uncertain	Developing country	366	21.22	0.189	0.320	GA	Other	BSI	6
Arpaci et al. [51]	Uncertain	Developing country	834	22.16	0.270	0.308	GA	BSMAS	STAI	6
Astolfi Cury et al. [16]	Before COVID-19	Developing country	100	33.71	0.370	0.160	GA	Other	HADS-A	2
Astolfi Cury et al. [16]	Before COVID-19	Developing country	100	34.98	0.250	0.360	GA	Other	HADS-A	2
Balta et al. [17]	Uncertain	Developing country	423	17.15	0.470	0.180	GA	Other	STAI	5
Brailovskaia and Margraf [52]	Before COVID-19	Developed country	179	22.52	0.229	0.320	GA	BSMAS	DASS-21-A	8
Brailovskaia and Margraf [53]	During COVID-19	Developed country	550	27.08	0.238	0.316	GA	BSMAS	DASS-21-A	9
Brailovskaia and Margraf [54]	During COVID-19	Developing country	1030	NA	0.555	0.382	GA	BSMAS	DASS-21-A	6
Brailovskaia and Margraf [54]	During COVID-19	Developed country	1012	NA	0.451	0.466	GA	BSMAS	DASS-21-A	6
Brailovskaia and Margraf [54]	During COVID-19	Developed country	1175	NA	0.475	0.467	GA	BSMAS	DASS-21-A	6
Brailovskaia and Margraf [54]	During COVID-19	Developed country	1012	NA	0.463	0.462	GA	BSMAS	DASS-21-A	6
Brailovskaia and Margraf [54]	During COVID-19	Developing country	1020	NA	0.485	0.472	GA	BSMAS	DASS-21-A	6
Brailovskaia and Margraf [54]	During COVID-19	Developed country	997	NA	0.481	0.477	GA	BSMAS	DASS-21-A	6
Brailovskaia and Margraf [54]	During COVID-19	Developed country	1022	NA	0.483	0.515	GA	BSMAS	DASS-21-A	6
Brailovskaia and Margraf [54]	During COVID-19	Developed country	1073	NA	0.446	0.300	GA	BSMAS	DASS-21-A	6
Brailovskaia and Margraf [54]	During COVID-19	Developed country	1077	NA	0.487	0.622	GA	BSMAS	DASS-21-A	6
Brailovskaia et al. [13]	Uncertain	Developed country	327	23.57	0.272	0.730	GA	BSMAS	DASS-21-A	6
Brailovskaia et al. [55]	During COVID-19	Developing country	1123	24.84	0.000	0.360	GA	BSMAS	DASS-21-A	4
Brailovskaia et al. [55]	During COVID-19	Developing country	1414	36.62	0.000	0.280	GA	BSMAS	DASS-21-A	4
Chang et al. [56]	Before COVID-19	Developing country	645	20.95	0.410	0.150	GA	BSMAS	HADS-A	7
Charzynska et al. [57]	Uncertain	Developed country	1157	20.33	0.472	0.260	GA	BSMAS	Other	5
Chen et al. [58]	During COVID-19	Developing country	2026	10.71	0.501	0.355	GA	BSMAS	DASS-21-A	6
Da Veiga et al. [59]	Before COVID-19	Developed country	404	21.65	0.267	0.230	GA	BSMAS	BSI	7
Dadiotis et al. [60]	Uncertain	Developed country	325	21.6	0.182	0.210	GA	BSMAS	DASS-21-A	2
Fekih Romdhane et al. [61]	During COVID-19	Developing country	700	21.5	0.324	0.307	GA	BSMAS	DASS-21-A	7
Flynn et al. [62]	Before COVID-19	Uncertain	717	31	0.191	0.334	GA	Other	DASS-21-A	5
Fung et al. [63]	Before COVID-19	Developing country	489	11.6	0.510	0.311	GA	BSMAS	DASS-21-A	7
Gao et al. [24]	Uncertain	Developing country	849	19	0.531	0.205	GA	Other	DASS-21-A	7
Gonzalez-Nuevo et al. [64]	During COVID-19	Developed country	1003	42.33	0.245	0.310	GA	Other	HADS-A	8
Hou et al. [65]	Before COVID-19	Developing country	641	19.9	0.256	0.220	GA	Other	STAI	6
Hussain and Griffiths [9]	Uncertain	Uncertain	638	32.08	0.524	0.380	GA	BSMAS	DASS-21-A	5
Hussain and Wegmann [66]	Uncertain	Uncertain	458	32.35	0.496	0.399	GA	BSMAS	Other	8
Imani et al. [67]	During COVID-19	Developing country	288	52.26	NA	0.340	GA	BSMAS	HADS-A	2
Islam et al. [68]	During COVID-19	Developing country	5511	21.2	0.589	0.542	GA	BSMAS	GAD	6
Islam et al. [69]	During COVID-19	Developing country	428	16.13	0.909	0.387	GA	BSMAS	GAD	5
Jahan et al. [70]	During COVID-19	Developing country	601	NA	0.572	0.460	GA	BSMAS	GAD	8
Jiang [71]	During COVID-19	Developing country	2056	NA	0.397	0.390	GA	Other	GAD	6
Jiang [71]	During COVID-19	Developing country	1067	NA	0.693	0.320	GA	Other	GAD	6
Kim et al. [72]	Uncertain	Developed country	209	NA	0.852	0.200	GA	Other	Other	8
Koc and Gulyagci [73]	Before COVID-19	Developing country	447	21.64	0.776	0.230	GA	Other	Other	6
Lin et al. [74]	Before COVID-19	Developing country	1073	36.57	0.428	0.170	GA	BSMAS	HADS-A	7
Lin, Imani et al. [75]	Uncertain	Developing country	1791	27.2	0.301	0.310	GA	BSMAS	HADS-A	7
Lozano Blasco et al. [76]	Before COVID-19	Developed country	361	NA	0.125	0.232	GA	Other	Other	5
Malak et al. [25]	Before COVID-19	Developing country	510	21.38	0.314	0.347	GA	Other	Other	7
Marino et al. [77]	During COVID-19	Developed country	726	28.59	0.482	0.350	GA	Other	DASS-21-A	7
Meshi and Ellithorpe [78]	During COVID-19	Developed country	403	20.25	0.367	0.290	GA	BSMAS	Other	8
Mitropoulou et al. [79]	During COVID-19	Developed country	255	27	0.310	0.180	GA	BSMAS	DASS-21-A	5
Ozimek et al. [80]	Uncertain	Developed country	1230	28.13	0.217	0.440	GA	BSMAS	DASS-21-A	6
Phillips and Wisniewski [81]	During COVID-19	Developed country	300	34.9	0.227	0.280	GA	BSMAS	DASS-21-A	4
Reer et al. [82]	Before COVID-19	Developed country	1929	27.77	0.488	0.431	GA	Other	GAD	9
Satici et al. [83]	Uncertain	Developing country	334	20.71	0.359	0.300	GA	Other	DASS-21-A	9
Sediri et al. [84]	During COVID-19	Developing country	751	37	0.000	0.390	GA	BSMAS	DASS-21-A	3
Shabahang et al. [85]	During COVID-19	Developing country	352	16.38	0.233	0.470	GA	BSMAS	BSI	7
Sotero et al. [86]	Before COVID-19	Developed country	403	22.25	0.345	0.180	GA	BSMAS	BSI	3
Stockdale and Coyne [87]	Before COVID-19	Uncertain	385	18.01	0.470	0.240	GA	Other	Other	8
Wang et al. [88]	Uncertain	Developing country	916	19.57	0.472	0.495	GA	BSMAS	DASS-21-A	6
White-Gosselin and Poulin [89]	Uncertain	Developed country	435	19.17	0.626	0.350	GA	BSMAS	Other	6
Wong et al. [90]	Before COVID-19	Developing country	300	20.89	0.407	0.344	GA	BSMAS	DASS-21-A	9
Yam et al. [91]	Before COVID-19	Developing country	307	21.64	0.324	0.190	GA	BSMAS	HADS-A	5
Yuan and Zhong [92]	During COVID-19	Developing country	1158	NA	0.444	0.172	GA	Other	Other	3
Yurdagul et al. [93]	Uncertain	Developing country	491	15.92	0.413	0.220	GA	BSMAS	STAI	6
Zhang et al. [94]	During COVID-19	Developing country	50,855	14.45	0.497	0.471	GA	BSMAS	GAD	6
Zhang, Wu et al. [95]	During COVID-19	Developing country	519	19.39	0.459	0.370	GA	Other	DASS-21-A	8
Zhang and Fan [96]	Uncertain	Developing country	286	20.32	0.423	0.612	GA	Other	DASS-21-A	9
Zhao et al. [97]	Uncertain	Developing country	931	19.59	0.450	0.210	GA	Other	STAI	8
Zhao, Zhou et al. [98]	During COVID-19	Developing country	60	NA	NA	0.280	GA	BSMAS	GAD	7
Apaolaza et al. [22]	Before COVID-19	Uncertain	346	18.73	0.483	0.440	SA	Other	IAS	6
Atroszko et al. [99]	Before COVID-19	Developed country	1157	20.33	0.472	0.190	SA	BSMAS	LSAS	8
Boursier et al. [18]	Before COVID-19	Developed country	578	16.1	0.375	0.182	SA	BSMAS	Other	5
Chen et al. [100]	Uncertain	Developing country	437	24.21	0.295	0.290	SA	Other	SIAS	8
Chen et al. [101]	Uncertain	Developing country	458	NA	0.354	0.290	SA	FAS	SAS-SMU	9
Chentsova et al. [102]	Uncertain	Uncertain	8912	20.25	0.292	0.300	SA	BSMAS	Other	2
Chu et al. [103]	Before COVID-19	Developing country	1401	18.83	0.585	0.390	SA	Other	SIAS	7
de Bérail et al. [20]	Before COVID-19	Uncertain	932	21.25	0.272	0.320	SA	Other	LSAS	8
Dempsey et al. [104]	Before COVID-19	Uncertain	291	20.03	0.424	0.300	SA	BSMAS	SIAS	6
Durak and Seferoglu [105]	Before COVID-19	Developing country	580	22.9	0.402	0.372	SA	Other	LSAS	8
Ekinci and Akat [106]	Uncertain	Developing country	508	NA	0.346	0.420	SA	Other	Other	6
Foroughi et al. [107]	Uncertain	Developing country	364	NA	0.489	0.495	SA	BSMAS	SAS-A	8
He [108]	Uncertain	Developing country	314	NA	0.522	0.650	SA	Other	IAS	7
Hu [109]	Uncertain	Developing country	645	13.17	0.526	0.350	SA	FAS	SASS-CS	7
Jia [110]	Uncertain	Developing country	605	NA	0.441	0.450	SA	FAS	SAS-A	7
Kim and Bae [14]	Uncertain	Developed country	377	22.42	0.448	0.771	SA	Other	SIAS	8
Lee-Won et al. [111]	Before COVID-19	Developed country	243	19.69	0.284	0.180	SA	FAS	SASS-CS	4
Li [112]	Uncertain	Developing country	825	NA	0.438	0.350	SA	Other	SAS-A	5
Lin et al. [113]	Before COVID-19	Developing country	254	NA	0.548	0.420	SA	Other	SAS-A	4
Liu [114]	Uncertain	Developing country	600	NA	0.462	0.220	SA	BSMAS	SAS-A	7
Lyvers et al. [115]	Uncertain	Developed country	217	22.33	0.203	0.220	SA	Other	SIAS	6
Majid et al. [116]	Before COVID-19	Developing country	378	NA	0.299	0.300	SA	Other	SIAS	6
Marino et al. [11]	During COVID-19	Developed country	756	28.74	0.496	0.540	SA	GPIUS2	SAS-SMU	6
Mou et al. [117]	During COVID-19	Developing country	2661	19.97	0.433	0.369	SA	BSMAS	LSAS	9
Naidu et al. [21]	Uncertain	Uncertain	1067	NA	0.446	0.795	SA	BSMAS	SIAS	3
Ruggieri et al. [118]	Uncertain	Uncertain	152	43.7	0.000	0.203	SA	GPIUS2	SAS-SMU	4
Ruggieri et al. [118]	Uncertain	Uncertain	152	13.7	0.573	0.330	SA	GPIUS2	SAS-SMU	4
Ruiz et al. [119]	Uncertain	Developed country	439	15.63	0.358	0.270	SA	Other	Other	8
She et al. [120]	Before COVID-19	Developing country	26,612	NA	0.563	0.296	SA	FAS	SASS-CS	9
Stănculescu [121]	During COVID-19	Developing country	705	30.24	0.390	0.210	SA	BSMAS	IAS	9
Teng et al. [122]	Uncertain	Developing country	970	18.92	0.269	0.249	SA	BSMAS	IAS	9
Tong [123]	Before COVID-19	Developing country	2872	NA	0.352	0.295	SA	PMSMUAQ	IAS	8
Tu et al. [124]	Uncertain	Developing country	649	14.48	0.515	0.640	SA	Other	SASS-CS	10
Wang [125]	Uncertain	Developing country	829	NA	0.474	0.390	SA	PMSMUAQ	IAS	7
Wang [126]	Uncertain	Developing country	1017	16.09	0.496	0.580	SA	PMSMUAQ	SAS-A	8
Wegmann et al. [127]	Before COVID-19	Developed country	334	19.27	0.284	0.455	SA	Other	Other	6
Yang [128]	Before COVID-19	Developing country	733	20	0.547	0.180	SA	Other	IAS	4
Yang [129]	Before COVID-19	Developing country	188	29.6	0.378	0.379	SA	Other	IAS	5
Yurdagul et al. [93]	Uncertain	Developing country	491	15.92	0.413	0.280	SA	BSMAS	SAS-A	7
Zhang [130]	Uncertain	Developing country	575	NA	0.490	0.521	SA	PMSMUAQ	SAS-A	8
Zhang et al. [131]	Uncertain	Developing country	2672	NA	0.417	0.290	SA	PMSMUAQ	IAS	6
Zhang [132]	Before COVID-19	Developing country	554	NA	0.334	0.583	SA	GPIUS2	Other	6
Zhao, Zhou et al. [98]	During COVID-19	Developing country	60	NA	NA	0.380	SA	BSMAS	IAS	7
Zhu [133]	Before COVID-19	Developing country	500	21.02	0.406	0.349	SA	Other	IAS	4
Arikan et al. [50]	Uncertain	Developing country	366	21.22	0.189	0.320	AA	Other	ECR	6
Blackwell et al. [134]	Before COVID-19	Developed country	207	22.15	0.242	0.342	AA	BSMAS	ECR	6
Boustead and Flack [8]	Uncertain	Uncertain	188	31.95	0.310	0.460	AA	Other	ECR	6
Chen [135]	Before COVID-19	Developed country	314	23.37	0.618	0.270	AA	Other	ECR	7
Chen et al. [100]	Uncertain	Developing country	437	24.21	0.295	0.180	AA	Other	Other	8
Chen et al. [136]	Before COVID-19	Developing country	489	18.81	0.389	0.230	AA	Other	ECR	9
de Bérail et al. [20]	Before COVID-19	Uncertain	932	21.25	0.272	0.120	AA	Other	RQ	6
Demircioglu and Goncu-Kose [23]	Before COVID-19	Developing country	547	15.8	0.484	0.320	AA	Other	Other	7
Ekinci and Akat [106]	Uncertain	Developing country	508	NA	0.346	0.330	AA	Other	Other	5
Flynn et al. [62]	Before COVID-19	Uncertain	717	31	0.191	0.373	AA	Other	ECR	5
Li [137].	Uncertain	Developing country	949	19.34	0.405	0.410	AA	PMSMUAQ	ECR	5
Liu and Ma [138]	Before COVID-19	Developing country	463	19.94	0.257	0.390	AA	Other	ECR	6
Marino et al. [11]	During COVID-19	Developed country	756	28.74	0.496	0.250	AA	GPIUS2	RQ	4
Mo et al. [139]	During COVID-19	Developing country	761	21.71	0.277	0.598	AA	GPIUS2	Other	7
Teng [140]	Uncertain	Developing country	518	NA	0.355	0.500	AA	PMSMUAQ	ECR	6
Tobin and Graham [12]	Uncertain	Uncertain	283	27.79	0.160	0.250	AA	BSMAS	Other	3
Worsley et al. [141]	Before COVID-19	Uncertain	915	20.19	0.316	0.280	AA	BSMAS	RQ	4
Worsley, McIntyre et al. [19]	Before COVID-19	Developed country	1029	19.8	0.252	0.150	AA	BSMAS	RQ	4
Wu [142]	Uncertain	Developing country	300	NA	0.223	0.419	AA	PMSMUAQ	ECR	0
Xia [143]	Uncertain	Developing country	392	NA	1.000	0.450	AA	Other	ECR	8
Xia [143]	Uncertain	Developing country	385	NA	0.000	0.310	AA	Other	ECR	8
Young et al. [144]	Uncertain	Uncertain	124	30.58	0.202	0.570	AA	GPIUS2	ECR	6
Bakioğlu et al. [145]	During COVID-19	Uncertain	419	25.43	0.310	0.671	FoMO	BSMAS	Other	7
Bakioğlu et al. [145]	During COVID-19	Uncertain	419	25.43	0.310	0.529	FoMO	BSMAS	FoMOS	7
Bendayan and Blanca [146]	Before COVID-19	Developed country	567	29.09	0.381	0.350	FoMO	FIQ	FoMOS	4
Błachnio and Przepiórka [147]	Before COVID-19	Developed country	360	22.22	0.360	0.450	FoMO	FIQ	FoMOS	6
Blackwell et al. [134]	Before COVID-19	Developed country	207	22.15	0.242	0.560	FoMO	BSMAS	FoMOS	6
Boustead and Flack [8]	Uncertain	Uncertain	188	31.95	0.310	0.630	FoMO	Other	FoMOS	7
Casale et al. [148]	Before COVID-19	Developed country	579	22.39	0.454	0.470	FoMO	BSMAS	FoMOS	6
Chen et al. [149]	Uncertain	Developing country	1153	18.54	0.239	0.506	FoMO	PMSMUAQ	FoMOS	8
Cheng et al. [150]	Uncertain	Developing country	314	21.78	0.325	0.471	FoMO	FAS	FoMOS	8
Cui et al. [151]	Uncertain	Developing country	541	20.82	0.368	0.521	FoMO	Other	FoMOS	6
Dempsey et al. [104]	Before COVID-19	Uncertain	291	20.03	0.424	0.320	FoMO	BSMAS	FoMOS	6
Ding et al. [152]	Uncertain	Developing country	621	20.96	0.498	0.680	FoMO	Other	FoMOS	9
Fabris et al. [153]	Before COVID-19	Developed country	472	13.5	0.500	0.480	FoMO	BSMAS	FoMOS	5
Fang et al. [154]	Uncertain	Developing country	501	19.6	0.293	0.450	FoMO	FIQ	FoMOS	5
Gao [155]	Uncertain	Developing country	1044	NA	0.370	0.462	FoMO	PMSMUAQ	FoMOS	5
Gioia et al. [156]	During COVID-19	Developed country	487	29.85	0.407	0.520	FoMO	Other	FoMOS	7
Gori et al. [10]	Uncertain	Developed country	470	33.76	0.298	0.451	FoMO	BSMAS	FoMOS	5
Gu [157]	Uncertain	Developing country	288	20.85	0.340	0.326	FoMO	Other	FoMOS	4
Gugushvili et al. [158]	Uncertain	Uncertain	151	28.26	0.305	0.460	FoMO	BSMAS	FoMOS	6
Hou [159]	Uncertain	Developing country	330	NA	0.391	0.514	FoMO	Other	FoMOS-MSME	6
Hu et al. [160]	Uncertain	Developing country	442	18.69	0.457	0.321	FoMO	Other	FoMOS	7
Hu [161]	Uncertain	Developing country	1092	NA	0.328	0.420	FoMO	FAS	FoMOS	4
Jiang and Jin [162]	Before COVID-19	Developing country	1804	NA	0.437	0.420	FoMO	PMSMUAQ	FoMOS	8
Li [163]	Uncertain	Developing country	1081	15.02	0.488	0.747	FoMO	FAS	FoMOS	7
Li et al. [164]	During COVID-19	Developing country	728	19.14	0.411	0.720	FoMO	FAS	FoMOS	8
Li [165]	Uncertain	Developing country	667	14.91	0.442	0.390	FoMO	Other	FoMOS	8
Li et al. [166]	Uncertain	Developing country	454	NA	0.436	0.719	FoMO	SNATS	T-S FoMOS	7
Li, Y [167].	During COVID-19	Developing country	216	NA	NA	0.410	FoMO	Other	FoMOS	6
Li, Y [167].	During COVID-19	Developing country	749	NA	NA	0.320	FoMO	Other	FoMOS	6
Liu and Ma [138]	Before COVID-19	Developing country	463	19.94	0.257	0.560	FoMO	Other	FoMOS	6
Ma and Liu [168]	Before COVID-19	Developing country	493	18.8	0.321	0.420	FoMO	Other	FoMOS	10
Mao [169]	During COVID-19	Developing country	442	18.69	0.229	0.320	FoMO	Other	FoMOS	6
Mao [170].	Uncertain	Developing country	1084	NA	0.483	0.714	FoMO	SNATS	FoMOS	8
Mo et al. [139]	During COVID-19	Developing country	761	21.71	0.277	0.659	FoMO	Other	FoMOS-MSME	7
Moore and Craciun [171]	Uncertain	Developed country	156	NA	0.440	0.430	FoMO	Other	FoMOS	6
Müller et al. [6]	Uncertain	Developed country	226	22	0.403	0.503	FoMO	Other	T-S FoMOS	7
Niu [172]	Uncertain	Developing country	401	NA	0.481	0.410	FoMO	BSMAS	FoMOS	6
Ozimek et al. [80]	Uncertain	Developed country	1230	28.13	0.217	0.639	FoMO	BSMAS	FoMOS	6
Phillips and Wisniewski [81]	During COVID-19	Developed country	300	34.9	0.227	0.420	FoMO	BSMAS	FoMOS	4
Pi and Li [173]	Uncertain	Developing country	4630	20.4	0.493	0.420	FoMO	PMSMUAQ	FoMOS	8
Pontes et al. [174]	Before COVID-19	Uncertain	532	NA	0.354	0.680	FoMO	BSMAS	FoMOS	7
Quaglieri et al. [175]	Uncertain	Developed country	397	22	0.308	0.492	FoMO	BSMAS	FoMOS	5
Servidio et al. [176]	Uncertain	Developed country	405	22.11	0.282	0.350	FoMO	BSMAS	FoMOS	3
Sheldon et al. [177]	Before COVID-19	Uncertain	252	23.96	0.422	0.400	FoMO	BSMAS	FoMOS	4
Sheldon et al. [177]	Before COVID-19	Uncertain	247	21.98	0.422	0.430	FoMO	BSMAS	FoMOS	4
Sheldon et al. [177]	Before COVID-19	Uncertain	221	21.36	0.422	0.340	FoMO	BSMAS	FoMOS	4
Song [26]	Uncertain	Developing country	1117	NA	0.349	0.470	FoMO	Other	FoMOS	6
Sun et al. [178]	During COVID-19	Developing country	311	28.67	0.396	0.581	FoMO	BSMAS	FoMOS	8
Tang [179]	Uncertain	Developing country	420	23.21	0.357	0.782	FoMO	FIQ	FoMOS-MSME	6
Tomczyk and Selmanagic-Lizde [180]	Before COVID-19	Developing country	717	13	0.473	0.560	FoMO	BSMAS	FoMOS	6
Unal-Aydin et al. [181]	Uncertain	Developing country	300	21.1	0.440	0.260	FoMO	BSMAS	FoMOS	7
Uram and Skalski [182]	During COVID-19	Developed country	309	25.11	0.414	0.430	FoMO	BSMAS	FoMOS	6
Varchetta et al. [183]	Uncertain	Developed country	306	21.8	0.503	0.730	FoMO	BSMAS	FoMOS	5
Wang [184]	Uncertain	Developing country	2185	NA	0.288	0.579	FoMO	SNATS	FoMOS	5
Wang et al. [185]	Uncertain	Developing country	1238	14.71	0.448	0.350	FoMO	FIQ	FoMOS	7
Wang [125]	Uncertain	Developing country	829	NA	0.474	0.430	FoMO	PMSMUAQ	FoMOS-MSME	7
Wegmann et al. [186]	Before COVID-19	Uncertain	270	23.43	0.296	0.393	FoMO	Other	T-S FoMOS	2
Wegmann et al. [187]	During COVID-19	Developed country	719	50.11	0.517	0.526	FoMO	Other	T-S FoMOS	6
Wei [188]	Before COVID-19	Developing country	636	19.68	0.000	0.170	FoMO	FAS	FoMOS	7
Wei [188]	Before COVID-19	Developing country	526	19.68	1.000	0.430	FoMO	FAS	FoMOS	7
Wu [142]	Uncertain	Developing country	300	NA	0.223	0.673	FoMO	PMSMUAQ	FoMOS-MSME	0
Xia [143]	Uncertain	Developing country	392	NA	1.000	0.430	FoMO	FAS	FoMOS	8
Xia [143]	Uncertain	Developing country	385	NA	0.000	0.170	FoMO	FAS	FoMOS	8
Xiong [189]	Uncertain	Developing country	678	NA	0.375	0.516	FoMO	SNATS	FoMOS	8
Yan [190]	Uncertain	Developing country	866	NA	0.505	0.184	FoMO	Other	FoMOS	5
Yan [191]	During COVID-19	Developing country	400	NA	0.488	0.405	FoMO	BSMAS	FoMOS	8
Yang [192]	Uncertain	Developing country	339	33.48	0.507	0.649	FoMO	Other	T-S FoMOS	6
Yin et al. [193]	During COVID-19	Developing country	108	20.87	NA	0.470	FoMO	FIQ	FoMOS	8
Zhang [194]	Uncertain	Developing country	563	NA	0.520	0.370	FoMO	FAS	FoMOS	7
Zhang [195]	Uncertain	Developing country	393	NA	0.529	0.648	FoMO	PMSMUAQ	FoMOS-MSME	8
Zhang et al. [131]	Uncertain	Developing country	2672	NA	0.417	0.490	FoMO	PMSMUAQ	T-S FoMOS	6
Zhang, Chen et al. [196]	Before COVID-19	Developing country	405	19.22	0.469	0.330	FoMO	FAS	FoMOS	7
Zhang et al. [197]	Before COVID-19	Developing country	526	19.56	0.481	0.400	FoMO	Other	FoMOS	9
Zhao et al. [15]	During COVID-19	Developing country	1373	19.53	0.433	0.750	FoMO	PMSMUAQ	FoMOS	8
Zhou and Fang [198]	Uncertain	Developing country	501	19.6	0.293	0.450	FoMO	FIQ	FoMOS	4

Note: *N* = sample size; Age = average age; Gender = proportion of males; *r* = Pearson’s product-moment coefficient *r*; GA = generalized anxiety; SA = social anxiety; AA = attachment anxiety; FoMO = fear of miss out; BSMAS = Bergen Social Media Addiction Scale [199, 200]; FAS = Facebook Addiction Scale [73]; FIQ = Facebook Intrusion Questionnaire [201]; GPIUS2 = Generalized Problematic Internet Use Scale 2 [202]; PMSMUAQ = Problematic Mobile Social Media Usage Assessment Questionnaire [203]; SNATS = Social Network Addiction Tendency Scale [204]; BSI = Brief Symptom Inventory [205]; DASS-21-A = the anxiety subscale of the Depression Anxiety Stress Scales [206]; GAD = Generalized Anxiety Disorder [207]; HADS-A = the anxiety subscale of the Hospital Anxiety And Depression Scale [208]; STAI = State-Trait Anxiety Inventory [209, 210]; IAS = Interaction Anxiousness Scale [211]; LSAS = Liebowitz Social Anxiety Scale [212]; SAS-SMU = Social Anxiety Scale for Social Media Users [213]; SAS-A = Social Anxiety for Adolescents [214]; SASS-CS = Social Anxiety Subscale of the Self-Consciousness Scale [215]; SIAS = Social Interaction Anxiety Scale [216, 217]; ECR = Experiences in Close Relationship Scale [218, 219]; RQ = relationship questionnaire [220]; FoMOS = Fear of Missing Out Scale [221]; FoMOS-MSME = FoMO Measurement Scale in the Mobile Social Media Environment [222]; T-S FoMOS = Trait-State Fear of missing Out Scale [186]

## Results

### Sample characteristics

**Fig. 1:**
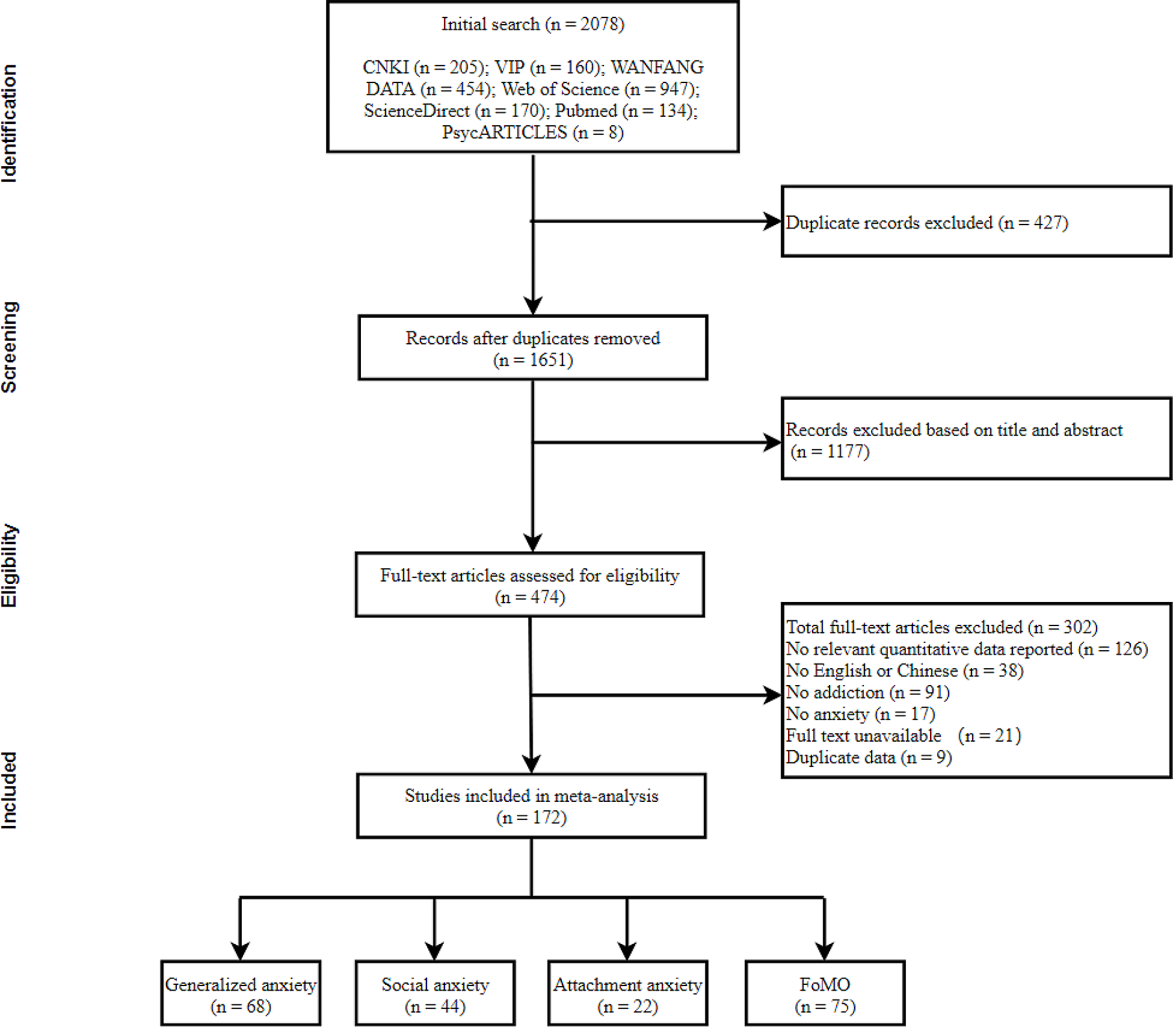
Flow chart of the search and selection strategy

The PRISMA search process is depicted in Fig. 1. The database search yielded 2078 records. After removing duplicate records and screening the title and abstract, the full text was subject to further evaluation. Ultimately, 172 records fit the inclusion criteria, including 209 independent effect sizes. The present meta-analysis included 68 studies on generalized anxiety, 44 on social anxiety, 22 on attachment anxiety, and 75 on fear of missing out. The characteristics of the selected studies are summarized in Table 2. The majority of the sample group were adults. Quality scores for selected studies ranged from 0 to 10, with only 34 effect sizes below the theoretical mean, indicating high quality for the included studies. The literature included utilized BSMAS as the primary tool to measure PSNU, DASS-21-A to measure GA, IAS to measure SA, ECR to measure AA, and FoMOS to measure FoMO.

### Overall analysis, homogeneity tests and publication bias

As shown in Table 3, there was significant heterogeneity between PSNU and all four anxiety symptoms (GA: *Q* = 1623.090, *I*^*2*^ = 95.872%; SA: *Q* = 1396.828, *I*^*2*^ = 96.922%; AA: *Q* = 264.899, *I*^*2*^ = 92.072%; FoMO: *Q* = 1847.110, *I*^*2*^ = 95.994%), so a random effects model was chosen. The results of the random effects model indicate a moderate positive correlation between PSNU and anxiety symptoms (GA: *r* = 0.350, 95% *CI* [0.323, 0.378]; SA: *r* = 0.390, 95% *CI* [0.347, 0.431]; AA: *r* = 0.345, 95% *CI* [0.286, 0.402]; FoMO: *r* = 0.496, 95% *CI* [0.461, 0.529]).

**Table 3:** Overall association between PSNU and anxiety symptoms

Anxiety type	Number Studies	Sample size	Effect size	95% CI for r		Test of null (two-tailed)		Homogeneity		
				Lower limit	Upper limit	*Z*-value	*p*-value	*Q*	*p*	*I* ^*2*^
Generalized anxiety	68	126,688	0.350/**0.388**	0.323/**0.362**	0.378/**0.413**	22.860	< 0.001	1623.090	< 0.001	95.872
Social anxiety	44	65,410	0.390/**0.437**	0.347/**0.395**	0.431/**0.478**	10.692	< 0.001	1396.828	< 0.001	96.922
Attachment anxiety	22	11,580	0.345	0.286	0.402	10.692	< 0.001	264.899	< 0.001	92.072
FoMO	75	48,659	0.496	0.461	0.529	23.610	< 0.001	1847.110	< 0.001	95.994

Note: The bolded indicates the coefficients corrected by the trim and fill method

**Fig. 2:**
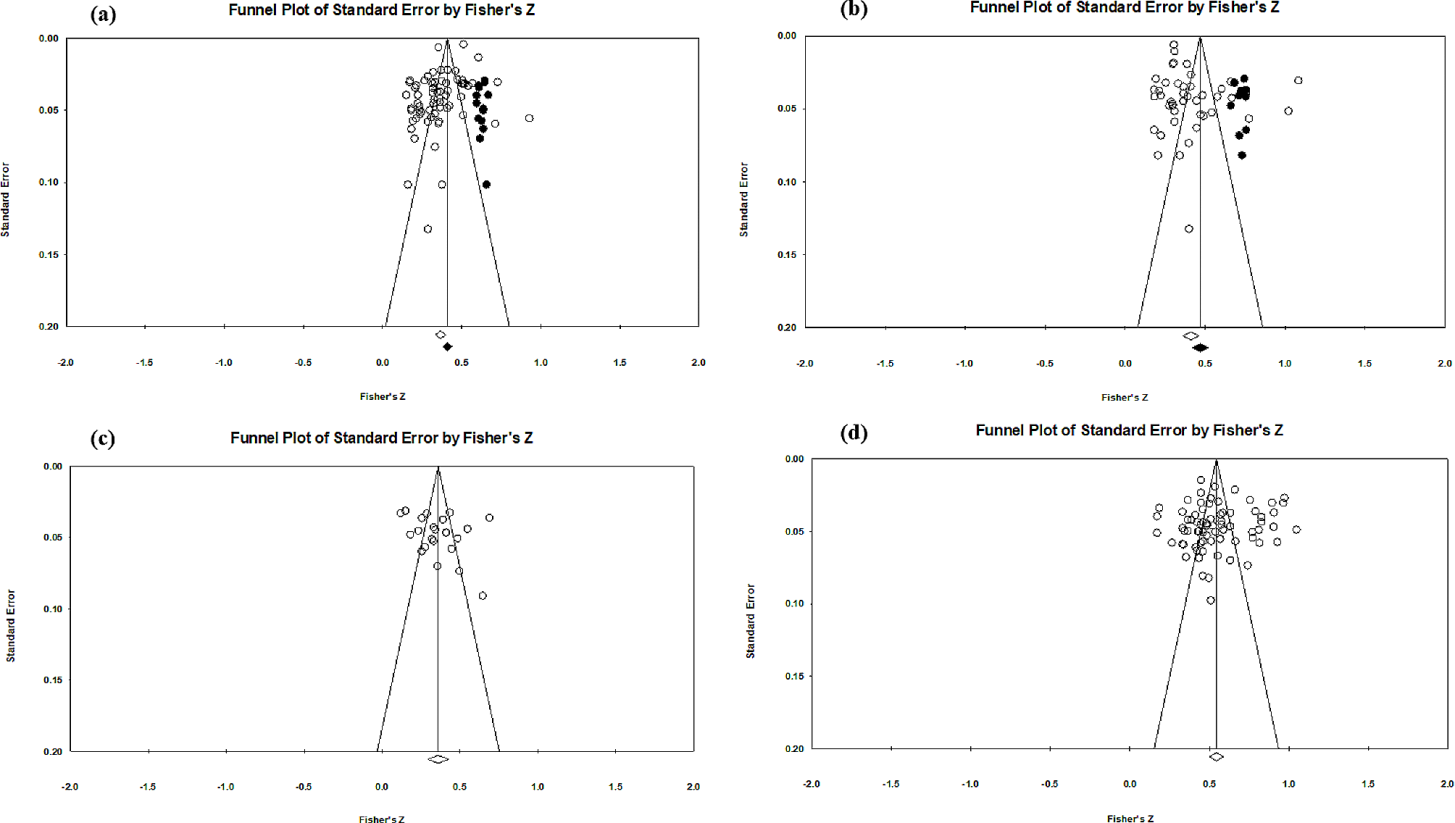
Funnel plot of the relationship between PSNU and anxiety symptoms. Note: Black dots indicated additional studies after using trim and fill method; (**a**) = Funnel plot of the PSNU and GA; (**b**) = Funnel plot of the PSNU and SA; (**c**) = Funnel plot of the PSNU and AA; (**d**) = Funnel plot of the PSNU and FoMO

Figure 2 shows the funnel plot of the relationship between PSNU and anxiety symptoms. No significant symmetry was seen in the funnel plot of the relationship between PSNU and GA and between PSNU and SA. And the Egger’s regression results also indicated that there might be publication bias (*t* = 3.775, *p* < 0.001; *t* = 2.309, *p* < 0.05). Therefore, it was necessary to use fail-safe number (Nfs) and the trim and fill method for further examination and correction. The Nfs for PSNU and GA as well as PSNU and SA are 4591 and 7568, respectively. Both Nfs were much larger than the standard 5*k* + 10. After performing the trim and fill method, 14 effect sizes were added to the right side of the funnel plat (Fig. 2.a), the correlation coefficient between PSNU and GA changed to (*r* = 0.388, 95% *CI* [0.362, 0.413]); 10 effect sizes were added to the right side of the funnel plat (Fig. 2.b), the correlation coefficient between PSNU and SA changed to (*r* = 0.437, 95% *CI* [0.395, 0.478]). The correlation coefficients did not change significantly, indicating that there was no significant publication bias associated with the relationship between PSNU and these two anxiety symptoms (GA and SA).

### Sensitivity analyses

Initially, the findings obtained through the one-study-removed approach indicated that the heterogeneities in the relationship between PSNU and anxiety symptoms were not attributed to any individual study. Nevertheless, it is important to note that sensitivity analysis should be performed based on literature quality [223] since low-quality literature could potentially impact result stability. In the relationship between PSNU and GA, the 10 effect sizes below the theoretical mean scores were excluded from analysis, and the sensitivity analysis results were recalculated (*r* = 0.402, 95% *CI* [0.375, 0.428]); In the relationship between PSNU and SA, the 8 effect sizes below the theoretical mean scores were excluded from analysis, and the sensitivity analysis results were recalculated (*r* = 0.431, 95% *CI* [0.387, 0.472]); In the relationship between PSNU and AA, the 5 effect sizes below the theoretical mean scores were excluded from analysis, and the sensitivity analysis results were recalculated (*r* = 0.367, 95% *CI* [0.298, 0.433]); In the relationship between PSNU and FoMO, the 11 effect sizes below the theoretical mean scores were excluded from analysis, and the sensitivity analysis results were recalculated (*r* = 0.508, 95% *CI* [0.470, 0.544]). The revised estimates indicate that meta-analysis results were stable.

### Moderator analysis

#### The impact of moderator variables on the relation between PSNU and GA

The results of subgroup analysis and meta-regression are shown in Table 4, the time of measurement significantly moderated the correlation between PSNU and GA (*Q*_*between*_ = 19.268, *df* = 2, *p* < 0.001). The relation between the two variables was significantly higher during the COVID-19 (*r* = 0.392, 95% *CI* [0.357, 0.425]) than before the COVID-19 (*r* = 0.270, 95% *CI* [0.227, 0.313]) or measurement time uncertain (*r* = 0.352, 95% *CI* [0.285, 0.415]).

The moderating effect of the PSNU measurement was significant (*Q*_*between*_ = 6.852, *df* = 1, *p* = 0.009). The relation was significantly higher when PSNU was measured with the BSMAS (*r* = 0.373, 95% *CI* [0.341, 0.404]) compared to others (*r* = 0.301, 95% *CI* [0.256, 0.344]).

The moderating effect of the GA measurement was significant (*Q*_*between*_ = 60.061, *df* = 5, *p* < 0.001). Specifically, when GA measured by the GAD (*r* = 0.398, 95% *CI* [0.356, 0.438]) and the DASS-21-A (*r* = 0.433, 95% *CI* [0.389, 0.475]), a moderate positive correlation was observed. However, the correlation was less significant when measured using the STAI (*r* = 0.232, 95% *CI* [0.187, 0.276]).

For the relation between PSNU and GA, the moderating effect of region, gender and age were not significant.

**Table 4:** Results of the moderating effects of PSNU and GA

Categorical variable	Q_between_ (df)	*p*-value	n of studies	r	95% CI	Continuous variable	b	SE	Z-value	95% CI	*p*
**Time of measurement**	19.268 (2)	< 0.001									
Before COVID-19			18	0.270	[0.227, 0.313]						
During COVID-19			32	0.392	[0.357, 0.425]						
Uncertain			18	0.352	[0.285, 0.415]						
**Region**	1.420 (2)	0.492									
Developed country			25	0.372	[0.326, 0.417]	**Gender**	0.089	0.105	0.846	[-0.121, 0.299]	0.401
Developing country			39	0.337	[0.296, 0.376]		*Q*_model_ (1, *df* = 64) = 0.716, *p* = 0.401				
Mixed			4	0.342	[0.280, 0.402]						
**PSNU measurement**	6.852(1)	0.009									
BSMAS			46	0.373	[0.341, 0.404]						
Others			22	0.301	[0.256, 0.344]						
**GA measurement**	60.061(5)	< 0.001				**Age**	-0.001	0.003	-0.249	[-0.006, 0.004]	0.804
DASS-21-A			31	0.398	[0.356, 0.438]		*Q*_model_ (1, *df* = 25) = 0.383, *p* = 0.804				
GAD			9	0.433	[0.389, 0.475]						
HADS-A			9	0.263	[0.205, 0.319]						
STAI			5	0.232	[0.187, 0.276]						
BSI			4	0.304	[0.172, 0.424]						
Others			10	0.273	[0.225, 0.321]						

#### The impact of moderator variables on the relation between PSNU and SA

The effects of the moderating variables in the relation between PSNU and SA were shown in Table 5. The results revealed a gender-moderated variances between the two variables (b = 0.601, 95% *CI* [ 0.041, 1.161], *Q*_model_ (1, k = 41) = 4.705, *p* = 0.036).

For the relation between PSNU and SA, the moderating effects of time of measurement, region, measurement of PSNU and SA, and age were not significant.

#### The impact of moderator variables on the relation between PSNU and AA

The effects of the moderating variables in the relation between PSNU and AA were shown in Table 6, region significantly moderated the correlation between PSNU and AA (*Q*_*between*_ = 6.410, *df* = 2, *p* = 0.041). The correlation between the two variables was significantly higher in developing country (*r* = 0.378, 95% *CI* [0.304, 0.448]) than in developed country (*r* = 0.242, 95% *CI* [0.162, 0.319]).

The moderating effect of the PSNU measurement was significant (*Q*_*between*_ = 6.852, *df* = 1, *p* = 0.009). Specifically, when AA was measured by the GPIUS-2 (*r* = 0.484, 95% *CI* [0.200, 0.692]) and the PMSMUAQ (*r* = 0.443, 95% *CI* [0.381, 0.501]), a moderate positive correlation was observed. However, the correlation was less significant when measured using the BSMAS (*r* = 0.248, 95% *CI* [0.161, 0.331]) and others (*r* = 0.313, 95% *CI* [0.250, 0.372]).

The moderating effect of the AA measurement was significant (*Q*_*between*_ = 17.283, *df* = 2, *p* < 0.001). The correlation was significantly higher when measured using the ECR (*r* = 0.386, 95% *CI* [0.338, 0.432]) compared to the RQ (*r* = 0.200, 95% *CI* [0.123, 0.275]).

For the relation between PSNU and AA, the moderating effects of time of measurement, region, gender, and age were not significant.

#### The impact of moderator variables on the relation between PSNU and FoMO

The effects of the moderating variables in the relation between PSNU and FoMO were shown in Table 7, the moderating effect of the PSNU measurement was significant (*Q*_*between*_ = 8.170, *df* = 2, *p* = 0.017). Among the sub-dimensions, the others was excluded because there was only one sample. Specifically, when measured using the FoMOS-MSME (*r* = 0.630, 95% *CI* [0.513, 0.725]), a moderate positive correlation was observed. However, the correlation was less significant when measured using the FoMOS (*r* = 0.472, 95% *CI* [0.432, 0.509]) and the T-S FoMOS (*r* = 0.557, 95% *CI* [0.463, 0.639]).

For the relationship between PSNU and FoMO, the moderating effects of time of measurement, region, measurement of PSNU, gender and age were not significant.

**Table 5:** Results of the moderating effects of PSNU and SA

Categorical variable	Q_between_ (df)	*p*-value	n of studies	r	95% CI	Continuous variable	b	SE	Z-value	95% CI	*p*
**Time of measurement**	4.394 (2)	0.111									
Before COVID-18			17	0.333	[0.291, 0.374]						
During COVID-19			4	0.381	[0.234, 0.511]						
Uncertain			23	0.429	[0.347, 0.504]						
**Region**	0.070 (2)	0.966									
Developed country			8	0.378	[0.196, 0.535]	**Gender**	0.601	0.277	2.169	[0.041, 1.161]	0.036
Developing country			29	0.385	[0.345, 0.424]		*Q*_model_ (1, *df* = 41) = 4.705, *p* < 0.05				
Mixed			7	0.413	[0.180, 0.602]						
**PSNU measurement**	4.989(4)	0.288									
BSMAS			12	0.350	[0.228, 0.461]						
FAS			5	0.322	[0.253, 0.388]						
GPIUS2			4	0.437	[0.281, 0.570]						
PMSMUAQ			5	0.421	[0.302, 0.526]						
Others			18	0.416	[0.337, 0.488]						
**SA measurement**	4.054(6)	0.669									
IAS			11	0.346	[0.282, 0.407]	**Age**	-0.004	0.006	-0.619	[-0.017, 0.009]	0.542
LSAS			4	0.314	[0.226, 0.397]		*Q*_model_ (1, *df* = 25) = 0.383, *p* = 0.542				
SAS-SMU			4	0.353	[0.168, 0.514]						
SAS-A			8	0.421	[0.324, 0.510]						
SASS-CS			4	0.384	[0.191, 0.549]						
SIAS			7	0.481	[0.232, 0.671]						
Others			6	0.375	[0.265, 0.475]						

**Table 6:** Results of the moderating effects of PSNU and AA

Categorical variable	Q_between_ (df)	*p*-value	n of studies	r	95% CI	Continuous variable	b	SE	Z-value	95% CI	*p*
**Time of measurement**	5.633 (2)	0.060									
Before COVID-18			9	0.274	[0.205, 0.340]						
During COVID-19			2	0.440	[0.047, 0.716]						
Uncertain			11	0.382	[0.318, 0.442]						
**Region**	6.410 (2)	0.041				**Gender**	0.057	0.167	0.339	[-0.292, 0.406]	0.738
Developed country			4	0.242	[0.162, 0.319]		*Q*_model_ (1, *df* = 20) = 0.115, *p* = 0.738				
Developing country			12	0.378	[0.304, 0.448]						
Mixed			6	0.339	[0.222, 0.446]						
**PSNU measurement**	16.837(3)	< 0.001									
BSMAS			4	0.248	[0.161, 0.331]						
GPIUS2			3	0.484	[0.200, 0.692]						
PMSMUAQ			3	0.443	[0.381, 0.501]	**Age**	0.009	0.008	1.042	[-0.009, 0.026]	0.314
Others			12	0.313	[0.250, 0.372]		*Q*_model_ (1, *df* = 15) = 1.086, *p* = 0.314				
**AA measurement**	17.283(2)	< 0.001									
ECR			13	0.386	[0.338, 0.432]						
RQ			4	0.200	[0.123, 0.275]						
Others			5	0.347	[0.168, 0.504]						

**Table 7:** Results of the moderating effects of PSNU and FoMO

Categorical variable	Q_between_ (df)	*p*-value	n of studies	r	95% CI	Continuous variable	b	SE	Z-value	95% CI	*p*
**Time of measurement**	5.343 (2)	0.069									
Before COVID-18			19	0.437	[0.381, 0.489]						
During COVID-19			15	0.531	[0.439, 0.613]						
Uncertain			41	0.509	[0.463, 0.552]						
**Region**	0.110 (2)	0.947									
Developed country			17	0.490	[0.435, 0.542]	**Gender**	0.102	0.161	0.633	[-0.219, 0.422]	0.529
Developing country			49	0.496	[0.450, 0.539]		*Q*_model_ (1, *df* = 70) = 0.401, *p* = 0.529				
Mixed			9	0.509	[0.402, 0.602]						
**PSNU measurement**	9.489(5)	0.091									
BSMAS			23	0.493	[0.438, 0.545]						
FAS			10	0.449	[0.290, 0.584]						
PMSMUAQ			9	0.544	[0.456, 0.620]						
FIQ			7	0.489	[0.346, 0.609]						
SNATS			4	0.639	[0.539, 0.722]	**Age**	0.003	0.005	0.689	[-0.006, 0.012]	0.494
Others			22	0.471	[0.409, 0.528]		*Q*_model_ (1, *df* = 50) = 0.474, *p* = 0.494				
**FoMO measurement**	8.130(2)	0.017									
FoMOS			62	0.472	[0.432, 0.509]						
FoMOS-MSME			6	0.630	[0.513, 0.725]						
T-S FoMOS			6	0.557	[0.463, 0.639]						

## Discussion

Through systematic review and meta-analysis, this study established a positive correlation between PSNU and anxiety symptoms (i.e., generalized anxiety, social anxiety, attachment anxiety, and fear of missing out), confirming a linear relationship and partially supporting the Social Cognitive Theory of Mass Communication [28] and the Cognitive Behavioral Model of Pathological Use [31]. Specifically, a significant positive correlation between PSNU and GA was observed, implying that GA sufferers might resort to social network for validation or as an escape from reality, potentially alleviating their anxiety. Similarly, the meta-analysis demonstrated a strong positive correlation between PSNU and SA, suggesting a preference for computer-mediated communication among those with high social anxiety due to perceived control and liberation offered by social network. This preference is often accompanied by maladaptive emotional regulation, predisposing them to problematic use. In AA, a robust positive correlation was found with PSNU, indicating a higher propensity for such use among individuals with attachment anxiety. Notably, the study identified the strongest correlation in the context of FoMO. FoMO’s significant association with PSNU is multifaceted, stemming from the real-time nature of social networks that engenders a continuous concern about missing crucial updates or events. This drives frequent engagement with social network, thereby establishing a direct link to problematic usage patterns. Additionally, social network’s feedback loops amplify this effect, intensifying FoMO. The culture of social comparison on these platforms further exacerbates FoMO, as users frequently compare their lives with others’ selectively curated portrayals, enhancing both their social networking usage frequency and the pursuit for social validation. Furthermore, the integral role of social network in modern life broadens FoMO’s scope, encompassing anxieties about staying informed and connected.

The notable correlation between FoMO and PSNU can be comprehensively understood through various perspectives. FoMO is inherently linked to the real-time nature of social networks, which cultivates an ongoing concern about missing significant updates or events in one’s social circle [221]. This anxiety prompts frequent engagement with social network, leading to patterns of problematic use. Moreover, the feedback loops in social network algorithms, designed to enhance user engagement, further intensify this fear [224]. Additionally, social comparison, a common phenomenon on these platforms, exacerbates FoMO as users continuously compare their lives with the idealized representations of others, amplifying feelings of missing out on key social experiences [225]. This behavior not only increases social networking usage but also is closely linked to the quest for social validation and identity construction on these platforms. The extensive role of social network in modern life further amplifies FoMO, as these platforms are crucial for information exchange and maintaining social ties. FoMO thus encompasses more than social concerns, extending to anxieties about staying informed with trends and dynamics within social networks [226]. The multifaceted nature of FoMO in relation to social network underscores its pronounced correlation with problematic social networking usage. In essence, the combination of social network’s intrinsic characteristics, psychological drivers of user behavior, the culture of social comparison, and the pervasiveness of social network in everyday life collectively make FoMO the most pronouncedly correlated anxiety type with PSNU.

Additionally, we conducted subgroup analyses on the timing of measurement (before COVID-19 vs. during COVID-19), measurement tools (for PSNU and anxiety symptoms), sample characteristics (participants’ region), and performed a meta-regression analysis on gender and age in the context of PSNU and anxiety symptoms. It was found that the timing of measurement, tools used for assessing PSNU and anxiety, region, and gender had a moderating effect, whereas age did not show a significant moderating impact.

Firstly, the relationship between PSNU and anxiety symptoms was significantly higher during the COVID-19 period than before, especially between PSNU and GA. However, the moderating effect of measurement timing was not significant in the relationship between PSNU and other types of anxiety. This could be attributed to the increased uncertainty and stress during the pandemic, leading to heightened levels of general anxiety [227]. The overuse of social network for information seeking and anxiety alleviation might have paradoxically exacerbated anxiety symptoms, particularly among individuals with broad future-related worries [228]. While the COVID-19 pandemic altered the relationship between PSNU and GA, its impact on other types of anxiety (such as SA and AA) may not have been significant, likely due to these anxiety types being more influenced by other factors like social skills and attachment styles, which were minimally impacted by the epidemic.

Secondly, the observed variance in the relationship between PSNU and AA across different economic contexts, notably between developing and developed countries, underscores the multifaceted influence of socio-economic, cultural, and technological factors on this dynamic. The amplified connection in developing countries may be attributed to greater socio-economic challenges, distinct cultural norms regarding social support and interaction, rising social network penetration, especially among younger demographics, and technological disparities influencing accessibility and user experience [229, 230]. Moreover, the role of social network as a coping mechanism for emotional distress, potentially fostering insecure attachment patterns, is more pronounced in these settings [231]. These findings highlight the necessity of considering contextual variations in assessing the psychological impacts of social network, advocating for a nuanced understanding of how socio-economic and cultural backgrounds mediate the relationship between PSNU and mental health outcomes [232]. Additionally, the relationship between PSNU and other types of anxiety (such as GA and SA) presents uniform characteristics across different economic contexts.

Thirdly, the significant moderating effects of measurement tools in the context of PSNU and its correlation with various forms of anxiety, including GA, and AA, are crucial in interpreting the research findings. Specifically, the study reveals that the Bergen Social Media Addiction Scale (BSMAS) demonstrates a stronger correlation between PSNU and GA, compared to other tools. Similarly, for AA, the Griffiths’ Problematic Internet Use Scale 2 (GPIUS2) and the Problematic Media Social Media Use Assessment Questionnaire (PMSMUAQ) show a more pronounced correlation with AA than the BSMAS or other instruments, but for SA and FoMO, the PSNU instrument doesn’t significantly moderate the correlation. The PSNU measurement tool typically contains an emotional change dimension. SA and FoMO, due to their specific conditional stimuli triggers and correlation with social networks [233, 234], are likely to yield more consistent scores in this dimension, while GA and AA may be less reliable due to their lesser sensitivity to specific conditional stimuli. Consequently, the adjustment effects of PSNU measurements vary across anxiety symptoms. Regarding the measurement tools for anxiety, different scales exhibit varying degrees of sensitivity in detecting the relationship with PSNU. The Generalized Anxiety Disorder Scale (GAD) and the Depression Anxiety Stress Scales 21 (DASS-21) are more effective in illustrating a strong relationship between GA and PSNU than the State-Trait Anxiety Inventory (STAI). In the case of AA, the Experiences in Close Relationships-21 (ECR-21) provides a more substantial correlation than the Relationship Questionnaire (RQ). Furthermore, for FoMO, the Fear of Missing Out Scale - Multi-Social Media Environment (FoMOS-MSME) is more indicative of a strong relationship with PSNU compared to the standard FoMOS or the T-S FoMOS. These findings underscore the importance of the selection of appropriate measurement tools in research. Different tools, due to their unique design, focus, and sensitivity, can reveal varying degrees of correlation between PSNU and anxiety disorders. This highlights the need for careful consideration of tool characteristics and their potential impact on research outcomes. It also cautions against drawing direct comparisons between studies without acknowledging the possible variances introduced by the use of different measurement instruments.

Fourthly, the significant moderating role of gender in the relationship between PSNU and SA, particularly pronounced in samples with a higher proportion of females. Women tend to engage more actively and emotionally with social network, potentially leading to an increased dependency on these platforms when confronting social anxiety [235]. This intensified use might amplify the association between PSNU and SA. Societal and cultural pressures, especially those related to appearance and social status, are known to disproportionately affect women, possibly exacerbating their experience of social anxiety and prompting a greater reliance on social network for validation and support [236]. Furthermore, women’s propensity to seek emotional support and express themselves on social network platforms [237] could strengthen this link, particularly in the context of managing social anxiety. Consequently, the observed gender differences in the relationship between PSNU and SA underscore the importance of considering gender-specific dynamics and cultural influences in psychological research related to social network use. In addition, gender consistency was observed in the association between PSNU and other types of anxiety, indicating no significant gender disparities.

Fifthly, the absence of a significant moderating effect of age on the relationship between PSNU and various forms of anxiety suggests a pervasive influence of social network across different age groups. This finding indicates that the impact of PSNU on anxiety is relatively consistent, irrespective of age, highlighting the universal nature of social network’s psychological implications [238]. Furthermore, this uniformity suggests that other factors, such as individual psychological traits or socio-cultural influences, might play a more crucial role in the development of anxiety related to social networking usage than age [239]. The non-significant role of age also points towards a potential generational overlap in social networking usage patterns and their psychological effects, challenging the notion that younger individuals are uniquely susceptible to the adverse effects of social network on mental health [240]. Therefore, this insight necessitates a broader perspective in understanding the dynamics of social network and mental health, one that transcends age-based assumptions.

### Limitations

There are some limitations in this research. First, most of the studies were cross-sectional surveys, resulting in difficulties in inferring causality of variables, longitudinal study data will be needed to evaluate causal interactions in the future. Second, considerable heterogeneity was found in the estimated results, although heterogeneity can be partially explained by differences in study design (e.g., Time of measurement, region, gender, and measurement tools), but this can introduce some uncertainty in the aggregation and generalization of the estimated results. Third, most studies were based on Asian samples, which limits the generality of the results. Fourth, to minimize potential sources of heterogeneity, some less frequently used measurement tools were not included in the classification of measurement tools, which may have some impact on the results of heterogeneity interpretation. Finally, since most of the included studies used self-reported scales, it is possible to get results that deviate from the actual situation to some extent.

## Conclusion

This meta-analysis aims to quantifies the correlations between PSNU and four specific types of anxiety symptoms (i.e., generalized anxiety, social anxiety, attachment anxiety, and fear of missing out). The results revealed a significant moderate positive association between PSNU and each of these anxiety symptoms. Furthermore, Subgroup analysis and meta-regression analysis indicated that gender, region, time of measurement, and instrument of measurement significantly influenced the relationship between PSNU and specific anxiety symptoms. Specifically, the measurement time and GA measurement tools significantly influenced the relationship between PSNU and GA. Gender significantly influenced the relationship between PSNU and SA. Region, PSNU measurement tools, and AA measurement tools all significantly influenced the relationship between PSNU and AA. The FoMO measurement tool significantly influenced the relationship between PSNU and FoMO. Regarding these findings, prevention interventions for PSNU and anxiety symptoms are important.

## Data Availability

The datasets are available from the corresponding author on reasonable request.

## Abbreviations

PSNU: Problematic social networking use
GA: Generalized anxiety
SA: Social anxiety
AA: Attachment anxiety
FoMO: Fear of miss out
BSMAS: Bergen Social Media Addiction Scale
FAS: Facebook Addiction Scale
FIQ: Facebook Intrusion Questionnaire
GPIUS2: Generalized Problematic Internet Use Scale 2
PMSMUAQ: Problematic Mobile Social Media Usage Assessment Questionnaire
SNATS: Social Network Addiction Tendency Scale
BSI: Brief Symptom Inventory
DASS-21-A: The anxiety subscale of the Depression Anxiety Stress Scales
GAD: Generalized Anxiety Disorder
HADS-A: The anxiety subscale of the Hospital Anxiety and Depression Scale
STAI: State-Trait Anxiety Inventory
IAS: Interaction Anxiousness Scale
LSAS: Liebowitz Social Anxiety Scale
SAS-SMU: Social Anxiety Scale for Social Media Users
SAS-A: Social Anxiety for Adolescents
SASS-CS: Social Anxiety Subscale of the Self-Consciousness Scale
SIAS: Social Interaction Anxiety Scale
ECR: Experiences in Close Relationship Scale
RQ: Relationship questionnaire
FoMOS: Fear of Missing Out Scale
FoMOS-MSME: FoMO Measurement Scale in the Mobile Social Media Environment
T-S FoMOS: Trait-State Fear of missing Out Scale
